# Evaluating large language models in pediatric fever management: a two-layer study

**DOI:** 10.3389/fdgth.2025.1610671

**Published:** 2025-09-03

**Authors:** Guijun Yang, Hejun Jiang, Shuhua Yuan, Mingyu Tang, Jing Zhang, Jilei Lin, Jiande Chen, Jiajun Yuan, Liebin Zhao, Yong Yin

**Affiliations:** ^1^ Department of Respiratory Medicine, Shanghai Children’s Medical Center, Shanghai Jiao Tong University School of Medicine, Shanghai, China; ^2^ Medical Department of Shanghai Children’s Medical Center, Shanghai Jiao Tong University School of Medicine, Shanghai, China; ^3^ Pediatric AI Clinical Application and Research Center, Shanghai Children’s Medical Center, Shanghai, China; ^4^ Shanghai Engineering Research Center of Intelligence Pediatrics (SERCIP), Shanghai, China; ^5^ Department of Respiratory Medicine, Hainan Branch of Shanghai Children’s Medical Center, Sanya Women and Children’s Hospital Affiliated to Hainan Medical College, Sanya, Hainan, China; ^6^ Department of Respiratory Medicine, Linyi Branch of Shanghai Children’s Medical Center, Linyi Maternal and Child Healthcare Hospital, Linyi, Shandong, China; ^7^ Shanghai Children’s Medical Center Pediatric Medical Complex (Pudong), Shanghai, China

**Keywords:** large language models, pediatric fever, medical communication, patient education, artificial intelligence in healthcare

## Abstract

**Background:**

Pediatric fever is a prevalent concern, often causing parental anxiety and frequent medical consultations. While large language models (LLMs) such as ChatGPT, Perplexity, and YouChat show promise in enhancing medical communication and education, their efficacy in addressing complex pediatric fever-related questions remains underexplored, particularly from the perspectives of medical professionals and patients’ relatives.

**Objective:**

This study aimed to explore the differences and similarities among four common large language models (ChatGPT3.5, ChatGPT4.0, YouChat, and Perplexity) in answering thirty pediatric fever-related questions and to examine how doctors and pediatric patients’ relatives evaluate the LLM-generated answers based on predefined criteria.

**Methods:**

The study selected thirty fever-related pediatric questions answered by the four models. Twenty doctors rated these responses across four dimensions. To conduct the survey among pediatric patients’ relatives, we eliminated certain responses that we deemed to pose safety risks or be misleading. Based on the doctors’ questionnaire, the thirty questions were divided into six groups, each evaluated by twenty pediatric relatives. The Tukey *post-hoc* test was used to check for significant differences. Some of pediatric relatives was revisited for deeper insights into the results.

**Results:**

In the doctors’ questionnaire, ChatGPT3.5 and ChatGPT4.0 outperformed YouChat and Perplexity in all dimensions, with no significant difference between ChatGPT3.5 and ChatGPT4.0 or between YouChat and Perplexity. All models scored significantly better in accuracy than other dimensions. In the pediatric relatives’ questionnaire, no significant differences were found among the models, with revisits revealing some reasons for these results.

**Conclusions:**

Internet searches (YouChat and Perplexity) did not improve the ability of large language models to answer medical questions as expected. Patients lacked the ability to understand and analyze model responses due to a lack of professional knowledge and a lack of central points in model answers. When developing large language models for patient use, it's important to highlight the central points of the answers and ensure they are easily understandable.

## 1 Introduction

Fever represents one of the most prevalent symptoms encountered in pediatric-related ailments. In England and Wales, pediatric patients seek guidance from general practitioners regarding fever-related conditions an average of 3.7 times annually (1). Fever, as a clinical manifestation, can broadly be categorized into two principal types: fever associated with infectious diseases and fever associated with non-infectious diseases (1, 2). The diseases that underlie fever are of significant concern, as they pose a considerable threat to the health and overall well-being of children. This concern is particularly pronounced in low-income and developing countries, where the impact of such illnesses can be especially severe (3, 4). Childhood fever frequently triggers parental anxiety, underscoring the need for healthcare professionals to address parents' concerns with clarity and empathy. Effective communication is essential for alleviating parental anxiety and fostering a positive doctor-patient relationship, contributing to a harmonious medical environment (5, 6).

In China, characterized by vast geographical and economic diversity, significant disparities in medical standards and resources particularly affect pediatric fever management and healthcare communication (7–9). Such disparities lead to significant variations in the clinical diagnosis, treatment capabilities, and patient communication skills among healthcare professionals across different regions. While current educational approaches for Chinese doctors, like clinical guideline learning and online self-study courses, provide some benefits, they often lack practical application and fail to address specific clinical queries, particularly in pediatric fever management. These methods offer certain advantages, including accessibility and up-to-date information. However, they also exhibit deficiencies, such as a lack of practical application and limitations in addressing specific clinical queries (10, 11).

The recent emergence of large language models (LLMs) such as ChatGPT, Perplexity, and YouChat etc. present a potential solution to these challenges, offering new avenues for medical communication and education. These advanced models, fine-tuned on vast textual datasets containing billions of parameters, possess the ability to generate text that closely resembles human language and excel in a diverse range of tasks. These tasks encompass answering inquiries, summarizing information, and fostering creative thinking (12). Recent investigations have scrutinized the efficacy of LLMs in responding to patient inquiries (13–16). John W. et al. found that ChatGPT's responses to patient-oriented questions were often superior to those of clinical doctors in quality and empathy (13). Conversely, Ashish S. and his research group identified potential inaccuracies in ChatGPT's responses, further exacerbated by the limitations imposed by the model's training data timeline, resulting in a divergence from the most current research advancements (14). These findings were echoed in a study conducted by Lingxuan Z. and team (15). A critical area for future research and development lies in broadening the scope to include comparative analyses across diverse large predictive models, thereby ensuring a comprehensive understanding of their operational variances and potential implications in clinical settings.

This research is meticulously designed to conduct an in-depth evaluation of four distinguished large language models—ChatGPT 3.5, ChatGPT 4.0, Perplexity, and YouChat—in their proficiency at addressing a diverse array of thirty complex pediatric febrile queries. The evaluation adopts a multi-dimensional approach, integrating insights from both seasoned medical professionals and patients to provide a holistic view. This study aims to bridge a critical gap in current academic research, offering advanced insights into the intricate roles that large language models play in the sphere of medical education and patient interaction. By thoroughly assessing these models' potential in streamlining clinical decision-making and enhancing patient engagement, the study aspires to significantly uplift the standard of medical care and strengthen the doctor-patient bond, ultimately contributing to the overall advancement of the healthcare industry.

## 2 Methods

### 2.1 Model selection

Based on previous research (15), user volume, and training methodologies, this study selected four models for investigation: ChatGPT3.5, ChatGPT4.0, YouChat, and perplexity. ChatGPT3.5 and ChatGPT4.0 were trained on a predefined dataset and did not connect to the internet post-launch. ChatGPT4.0 employs a more extensive and diverse pre-training dataset compared to ChatGPT3.5, along with more advanced training techniques, such as more effective model optimization algorithms and smarter parameter initialization methods. YouChat (the basic version) and Perplexity (the basic version), used in this research, were large language models developed based on GPT3.5. YouChat enhanced ChatGPT3.5 by incorporating an internet search function, while perplexity combines ChatGPT3.5 with a search engine to generate answers.

### 2.2 Question selection and answering with large language models

We selected thirty questions from “Thirty key issues on management of febrile children” published in the journal “Chinese Journal of Applied Clinical Pediatrics” for testing (Table 1). All questions were asked in Chinese and recorded in Chinese, and we have translated them into English for presentation. In the doctor's version of the questionnaire, for all models, the prompt was set as: “Assume you are an expert in the field of pediatrics, and the following questions are all related to pediatrics. Please answer the following questions in less than 500 words”. In the version of the questionnaire for relatives of pediatric patients, the prompt was set as: “Assume you are an expert in the field of pediatric medicine, and the following questions are all related to pediatric medicine. Please answer the following questions concisely and in an easy-to-understand manner in less than 200 words”. For all models, the questions were inputted in the exact same order and content. To evaluate the internal stability of the models, we created five dialogues using the same input method for questioning. The stability of the five responses was jointly evaluated by the project team members, and the results were recorded on a ten-point scale, with a minimum of 1 and a maximum of 10 (Supplementary Material S1).

**Table 1:** Questions used to test the performance of LLMs.

Thirty key issues on management of febrile children	
1	What degree of body temperature is a fever? Is fever good for the body?
2	Which method of measuring body temperature is considered most accurate? How frequently should body temperature be measured?
3	What are the common causes of acute fever in children?
4	What are the common pathogens causing fever due to upper respiratory tract infections? How can this be determined?
5	How can the severity of a fever in children be assessed
6	Can a high fever cause damage to the brain
7	Does a higher body temperature indicate a more severe illness?
8	Why is it important to consider both the degree of temperature increase and comfort level before deciding to use antipyretic analgesics? How can discomfort in a child with fever be identified?
9	Is it necessary to use antimicrobial drugs when a child has a fever?
10	Why is a complete blood count test commonly performed during fever? Is it true that a complete blood count test becomes accurate only after 24 h of fever onset?
11	How should a fever of unknown origin be managed
12	How should a child with a history of febrile seizures be managed in the event of another fever? How should an episode of a febrile seizure be handled?
13	Does administering intravenous fluids during high fever expedite recovery from the illness?
14	Can vaccination lead to fever? How should such a fever be managed? When is it necessary to visit a hospital?
15	In what circumstances should a lumbar puncture be performed on a child with fever? Is this a surgical procedure? Does it pose any risk of brain damage?
16	What dietary considerations should be taken into account during a fever?
17	How should home care be provided for a child with a fever?
18	At what temperature should antipyretic analgesics be used for a fever? How should these medications be administered?
19	If fever persists or recurs after using antipyretic analgesics, does it indicate that the medication is ineffective? Is it necessary to visit a hospital in such cases?
20	If there is no reduction in body temperature after taking antipyretic analgesics, is it safe to take an additional dose? Can antipyretic analgesics be administered via intramuscular or intravenous injection?
21	If vomiting occurs after taking antipyretic analgesics, is it necessary to administer another dose?
22	Can acetaminophen and ibuprofen be used alternately for fever reduction?
23	Is physical cooling an appropriate method for reducing fever in children? What are the different methods of physical cooling?
24	How should fever be managed in newborns and infants under three months of age?
25	During fever with chills, should physical cooling be continued, or should warmth be maintained?
26	Is it advisable to wear more clothes or cover with more blankets to induce sweating during a fever?
27	How should antipyretic analgesics be selected for a child with asthma who has a fever?
28	How should antipyretic analgesics be chosen in cases of fever accompanied by liver or kidney dysfunction?
29	How should antipyretic analgesics be selected for a fever in patients with hemorrhagic disorders?
30	How should antipyretic analgesics be used for a child with Kawasaki disease who has a fever?

### 2.3 Model evaluation dimensions

To evaluate the model results from the perspectives of both pediatric patients' relatives and doctors, we designed two versions of questionnaires: one for doctors and one for relatives of pediatric patients. In the doctor's version of the questionnaire, we adopted “accuracy”, “correctness”, “completeness”, and “practicality” to evaluate the responses from different models. “Accuracy” was defined as the degree of alignment between the response and the objective of the question, reflecting the model's ability to understand the user's query. “Correctness” referred to the degree of agreement between the response and reference answers or the clinical experience of the subjects in terms of objectivity and accuracy. “Completeness” was defined as the extent to which the response aligns with the reference answer and subjects' clinical experience in terms of thoroughness and completeness. “Practicality” was defined as the degree to which the response could be applied in daily diagnosis and treatment, reflecting the model's ability to solve practical problems. A ten-point scale was used for result recording. Responses like “I cannot answer” were scored 0, while other answers were scored between 1 and 10. Definitions of the four evaluation dimensions were placed on the first page of the questionnaire to clearly inform the subjects. In the pediatric patients' relatives' version of the questionnaire, we used four questions to evaluate the model's performance: “Does the answer match the question?”, “Is the answer easy to understand and clear?”, “Does the answer address my doubts?”, and “Does the answer make me feel comfortable?”

### 2.4 Questionnaire design

Each doctor version of the questionnaire contained thirty questions, arranged in the same order, with one reference answer provided by “Thirty key issues on management of febrile children” and four answers given by different large language models (Supplementary Material S2). The four responses under each question were presented in random order, and the participants were not informed which model corresponded to each answer. Before distributing the questionnaire version for the relatives of pediatric patients, we assessed all responses for safety reasons. Responses we deemed to pose safety risks or to be misleading were excluded (Supplementary Files) and scored as zero. To improve the quality of questionnaire completion, we divided the thirty questions based on the results of the doctor version to ensure that each participant could complete a questionnaire in just 5–15 min. Each questionnaire for the relatives of pediatric patients contained five questions, with the rest of the design being the same as the doctor version. Additionally, for the version aimed at the relatives, we conducted a pre-release test with non-medical parents, and modifications were made based on their feedback before the official release. We arranged the thirty questions of the physician version questionnaire in descending order of scores, grouping every six questions into a tier for stratified sampling without replacement, to ensure that each questionnaire for relatives of pediatric patients had a similar overall difficulty (Supplementary Files). The doctor version of the questionnaire was distributed in paper form to eligible doctors and was uniformly collected back on December 6, 2023. The questionnaire for the relatives of pediatric patients was distributed online in an electronic format on December 24, 2023, and was uniformly collected back on December 28, 2023. The flowchart of the study is presented in Supplementary Figure S1.

### 2.5 Participant inclusion

This study included doctors who met the following four criteria: (1). Hold a Master's degree in medicine or higher. (2). Below 60 years of age. (3). Have worked in the pediatrics department of a tertiary hospital for more than ten years. (4). Hold the professional title of attending physician or higher.

The relatives of pediatric patients included in the study had to meet the following three criteria: (1). Possess basic literacy skills. (2). Work in a non-medical industry. (3). Have at least one child of their own or have had close contact with a child for more than three months.

### 2.6 Questionnaire quality control

We implemented the following quality control measures for the doctor version of the questionnaire: a sample was deemed to have failed quality control if it exhibited five instances of quality control anomalies. Only samples that passed quality control were included in the analysis. The following situations were defined as one instance of a quality control anomaly: (1) Assigning a high score to a response that had obvious errors/deficiencies was considered one instance of a quality control anomaly. (2) If the questionnaire was completed in less than 2 h, it was counted as one quality control anomaly. (3) If there were scores for three responses that were clear outliers, it was counted as one quality control anomaly. If there were less than three such scores, it was counted as three.

A similar method was used for quality control of the questionnaire for the relatives of pediatric patients. To maintain a consistent sample size with the doctor version, the first twenty samples that met the quality control criteria were included for each type of questionnaire for the relatives of pediatric patients.

### 2.7 Data analysis

All data were analyzed using R version 4.3.2. To provide an overview of how the four large language models responded to questions about fever, we calculated the average score for all questions answered by four models, and the results were displayed in bar charts. Next, for each model, we calculated the average score for all evaluative dimensions of each question to analyze the specific responses of different models to each question. Sankey diagrams were used to illustrate the similarities and differences between the TOP5 and Bottom5 questions in terms of total scores answered by the four models. To compare whether there were differences between the models, we first calculated the average score for each question under different models, and conducted hypothesis testing using Tukey's *post hoc* test. We then used Tukey's *post hoc* test to compare whether there were differences in the performance of the four models across various dimensions. Finally, we compared whether there were differences between the various dimensions within each model, using Tukey's *post hoc* test to assess the significance of the differences.

## 3 Results

### 3.1 Evaluation of model stability

The researchers in this study scored the consistency of the answers as shown in Supplementary Table S1. The five researchers involved in the evaluation each gave a consistency score of over 8 for the five responses to thirty questions from the four models. The researchers selected the results of the second response by random sampling for subsequent studies.

### 3.2 Questionnaire distribution and recall

A total of 20 copies of the doctor's version of the questionnaire were distributed and all 20 were recalled, with 19 qualifying for quality control, yielding a qualification rate of 95%. For the relatives of pediatric patient's version, the first 20 of the recalled questionnaires in each group that passed quality control were analyzed.

### 3.3 Evaluation of LLMs' performance

**Figure 1:**
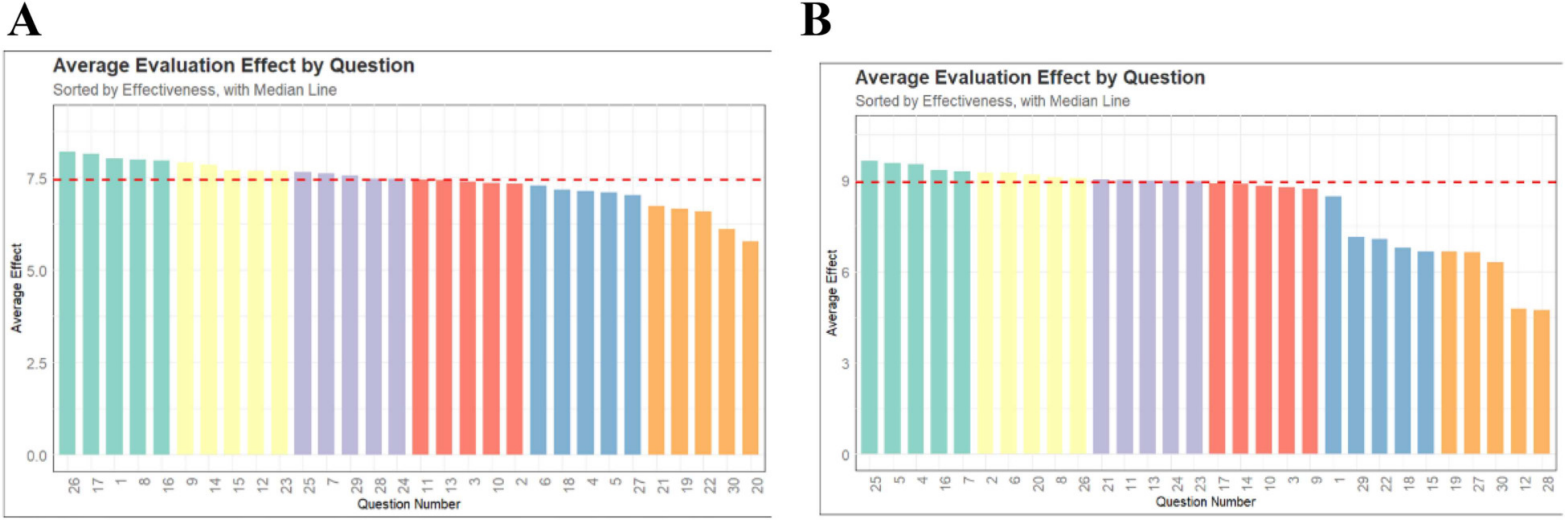
The scores of all models on each question. **(A)** The doctor's version of the questionnaire. **(B)** The pediatric patient's relative's version of the questionnaire.

Figure 1 displayed the response situation of LLMs to 30 questions. In the doctor's version of the questionnaire (Figure 1A), the median score for all questions answered by LLMs was 7.5, with the highest scores for questions 26, 17, 1, 8, and 16. The lowest scoring questions were 20, 30, 22, 19, and 21. In the relatives of pediatric patient's version of the questionnaire (Figure 1B), the median score for all questions answered by LLMs was 9, with the highest scores for questions 25, 5, 4, 16, and 7. The lowest scoring questions were 28, 12, 30, 27, and 19.

**Figure 2:**
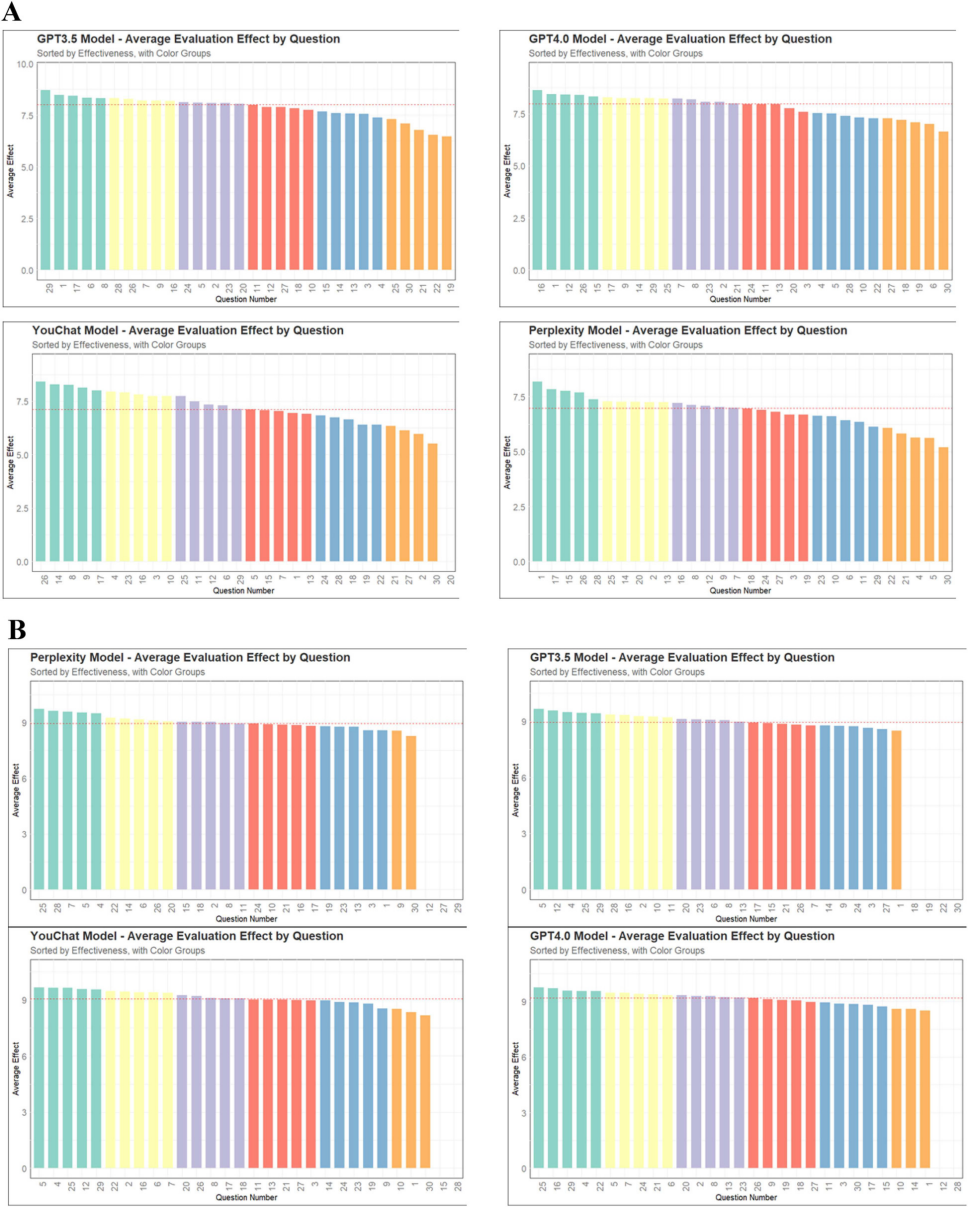
The scores of different models on each question. **(A)** The doctor's version of the questionnaire. **(B)** The pediatric patient's relative's version of the questionnaire.

**Figure 3:**
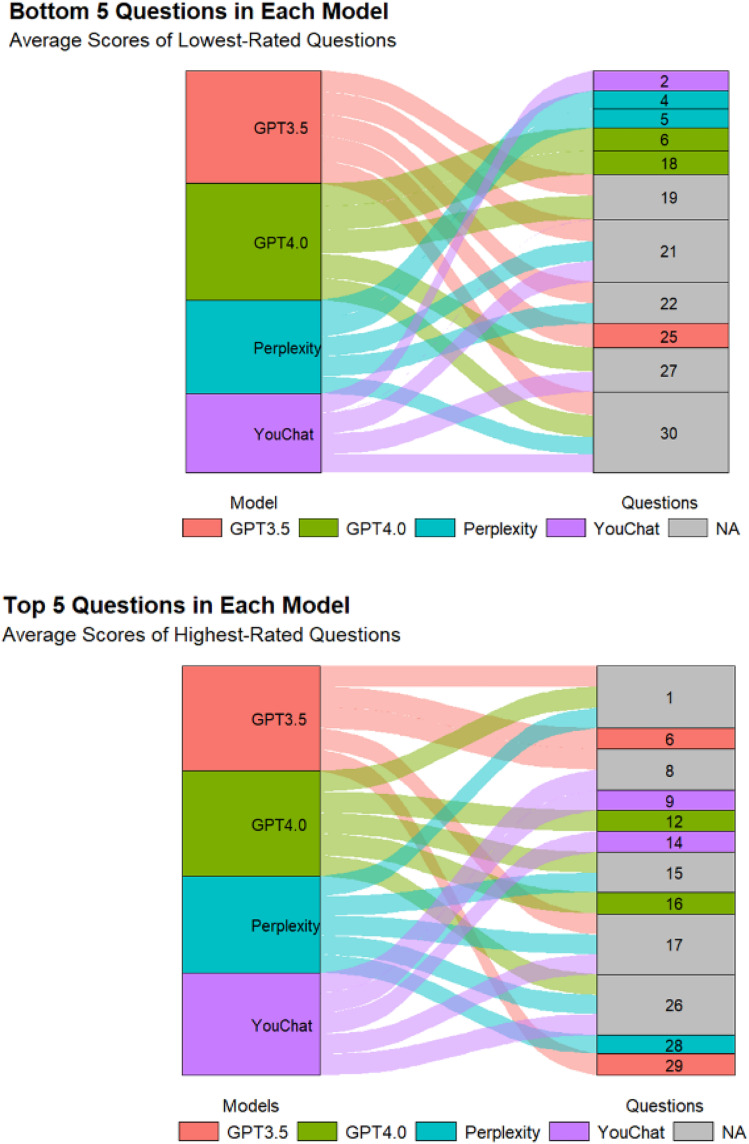
Sankey diagram of the Doctor's version questionnaire.

Figure 2 displayed the scores of different models on each question. In the doctor's version of the questionnaire (Figure 2A), ChatGPT3.5 and ChatGPT4.0 had higher median scores, while Perplexity and YouChat had lower median scores. Among the four models, only YouChat responded with “I don't know” to question 20. In the relatives of pediatric patient's version of the questionnaire (Figure 2B), the median scores of the four models were not much different. Responses by Perplexity to questions 12, 27, and 29 were excluded, as were YouChat's responses to questions 15 and 28. We also excluded responses of ChatGPT3.5 to questions 18, 19, 22, and 30, and ChatGPT4.0's responses to questions 12 and 28. Figure 3 showed the differences and similarities between the highest and lowest scoring questions among different models in the doctor's version of the questionnaire. It was found that multiple models performed well in answering questions 1, 8, 15, 17, and 26. However, in the questions with poorer responses, all models performed poorly in answering question 30, and multiple models had poorer responses to questions 19, 21, 22, and 27.

### 3.4 Comparison of answering effectiveness of different models

**Figure 4:**
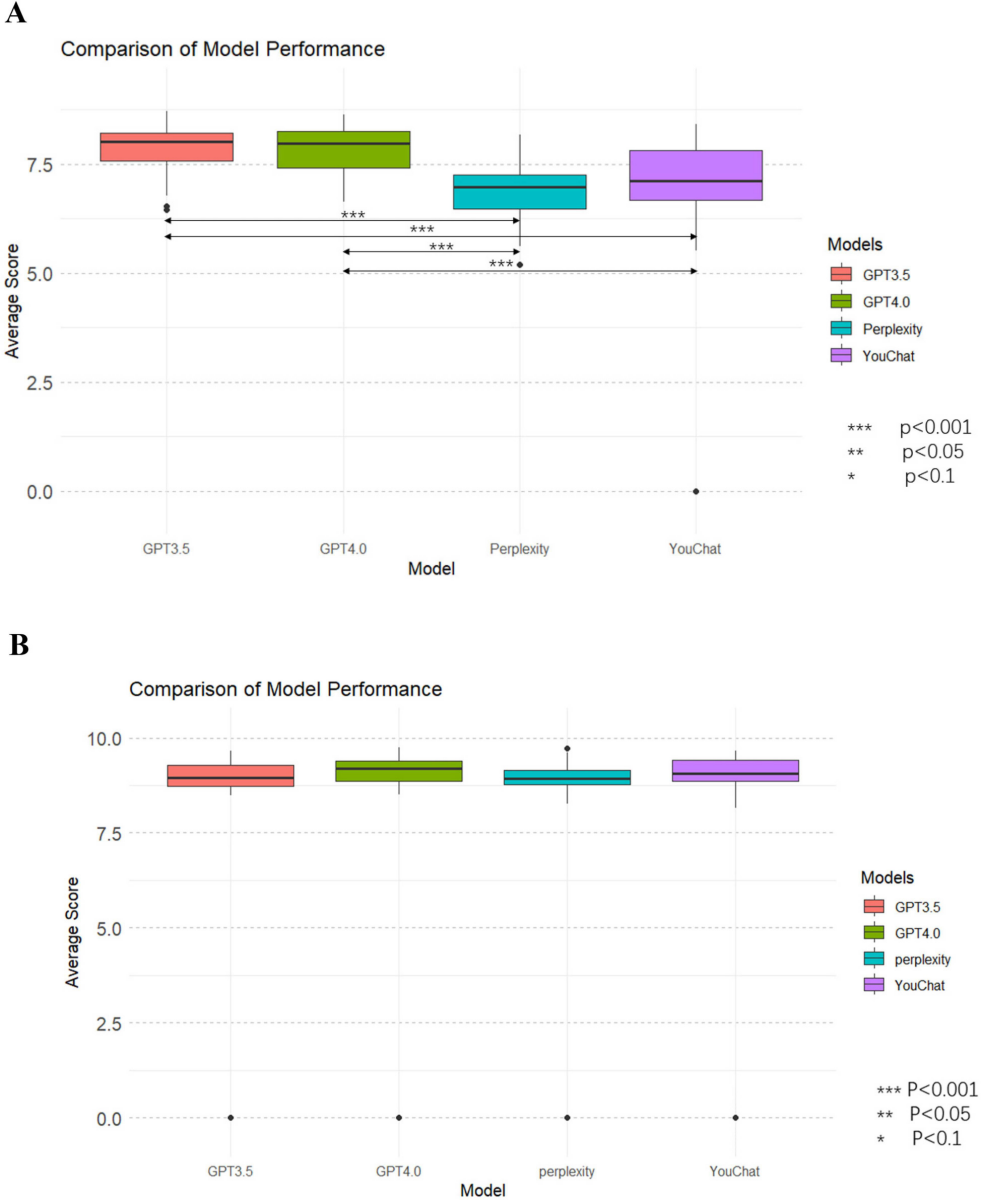
Average scores of all dimensions for each model and comparison of models. **(A)** The doctor's version **(B)** The pediatric patient's relative's version.

**Figure 5:**
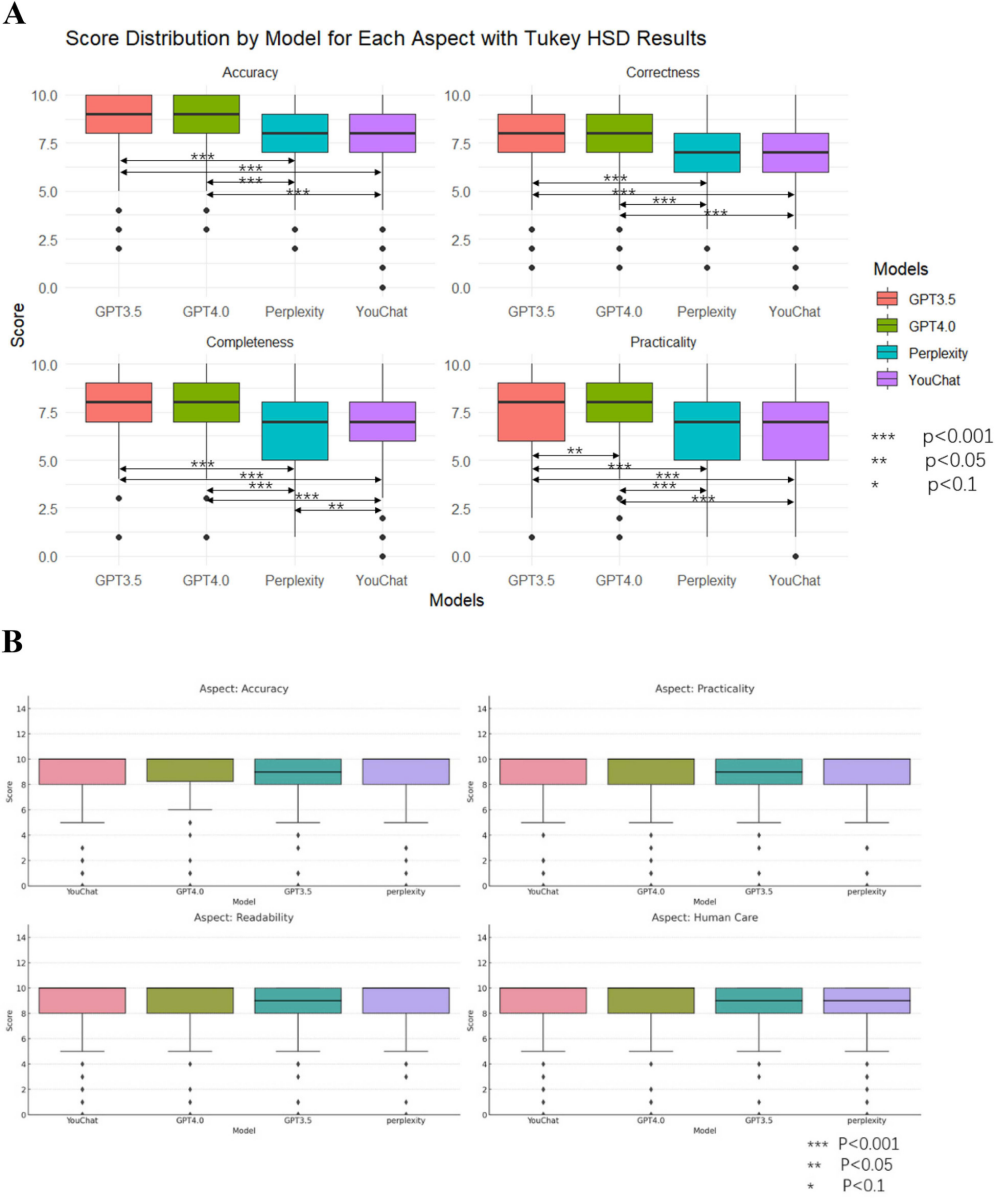
Scores and comparisons of different models under each dimension. **(A)** The doctor's version **(B)** The pediatric patient's relative's version.

**Figure 6:**
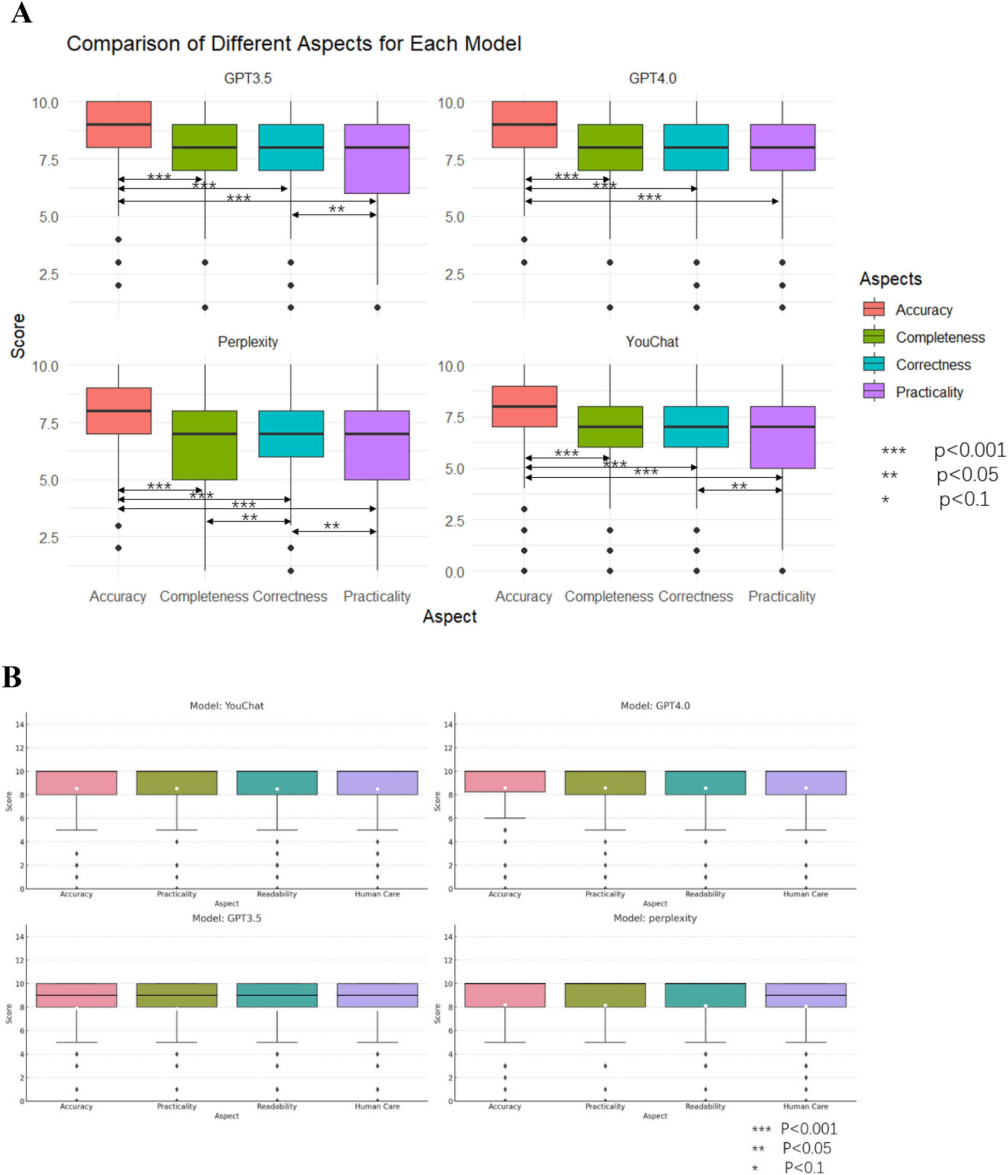
Scores and comparisons of different dimensions within each model. **(A)** The doctor's version **(B)** The pediatric patient's relative's version.

Figure 4 showed the average scores of each model on all questions. In the doctor's version of the questionnaire (Figure 4A), ChatGPT3.5 and ChatGPT4.0 had significantly outperformed Perplexity and YouChat, but there was no significant difference between ChatGPT3.5 and ChatGPT4.0, nor between YouChat and Perplexity. In the pediatric patient's relative's version of the questionnaire (Figure 4B), there was no significant difference among all the models. Subsequently, to assess whether there were differences between the models in each evaluation dimension, Figure 6 was drawn. In the results of the doctor's version questionnaire (Figure 5A), it was found that in terms of accuracy, ChatGPT3.5 and ChatGPT4.0 significantly outperformed Perplexity and YouChat, with no significant difference between ChatGPT3.5 and ChatGPT4.0, nor between Perplexity and YouChat. The same trend was observed in terms of correctness, completeness, and practicality. In the results of the pediatric patient's relative's version of the questionnaire (Figure 5B), it was found that there were no statistical differences in the scores of the four models across all dimensions.

### 3.5 Comparison in different dimensions of each model

Figure 6 showed the scores of each model in different dimensions. In the results of the doctor's version questionnaire (Figure 6A), it was found that each model scored significantly higher in accuracy than in all other dimensions. Furthermore, the Perplexity model's score in practicality was lower than in correctness, and its score in completeness was lower than in correctness. The YouChat model's score in practicality was lower than in correctness. In the results of the pediatric patient's relative's version (Figure 6B), no significant differences between dimensions were observed in any of the models.

## 4 Discussion

Large language models had shown tremendous potential in the medical field, such as assisting doctors in diagnosing diseases, helping patients and their families handle simple medical issues to alleviate pressure on the healthcare system, and aiding patients in better understanding medical problems. There were a few research and trials in this area. Although it had been reported that ChatGPT passed the U.S. medical licensing exam and performed well in some specialized medical question banks (17, 18), in the real world, medical questions are often open-ended and subjective, like “My child has a fever, what should I do?” Therefore, using large language models to take medical exams and simply evaluating their application value in medicine based on their scores was not an adequate method to assess their value. Moreover, due to significant differences in medical knowledge between doctors and patients, their evaluations of the same model could vary greatly. Thus, conducting dual-layer research involving both doctors and patients was essential in evaluating the application value of large language models in medicine.

In the doctor's version of the survey, we found that all four models exhibited a similar trend in both overall effectiveness and across the four dimensions, namely that ChatGPT3.5 and ChatGPT4.0 significantly outperformed YouChat and Perplexity, lending greater reliability to the results. However, in the pediatric patient's relative's version of the questionnaire, this trend was not observed, and many questionnaires scored a full 10 points, indicating a high proportion of invalid questionnaires. To explain this phenomenon, we revisited some of the subjects. From the revisits, we found: (1). Due to a lack of relevant medical knowledge, the relatives of pediatric patients struggled to judge the correctness of the answers provided by the large language models; (2). Even answers that appeared easy to understand to the researchers (doctors) were deemed too technical and abstruse by the relatives, making it difficult for them to fully read and evaluate the responses; (3). Despite the researchers providing prompts for the large language models to answer briefly, the responses were often lengthy and lacked a prominent central conclusion, making it difficult for non-medical professionals to grasp the main point. For instance, in question 28, “How should antipyretic analgesics be chosen in cases of fever accompanied by liver or kidney dysfunction?”, the large language model elaborated on the pros and cons of using acetaminophen and ibuprofen without telling the user which drug to choose. Therefore, in developing medical large language models for patient use, it is important to: (1). Ensure the answers are easy to understand, avoiding too many technical concepts and obscure descriptions; (2). Highlight the central conclusion, reduce related analyses appropriately, or prompt patients to ask follow-up questions if they wish to know more.

Most large language models on the market were based on GPT series pre-trained models with additional fine-tuning. A major issue with these models was the outdated knowledge base, which was not updated on time. A study had shown that ChatGPT could give incorrect answers due to reliance on outdated information (15). Some models had added search functions to overcome the limitations of outdated knowledge bases, but there were concerns about the decline in training data quality. This study found that ChatGPT3.5 and ChatGPT4.0, which do not perform internet searches, outperformed YouChat and Perplexity, which do. Internet searches did not enhance the models' answering capabilities as expected. One plausible explanation for the superior performance of ChatGPT3.5 and ChatGPT4.0, despite their lack of real-time internet connectivity, lies in the breadth and quality of their pre-training datasets, which incorporate a vast array of authoritative medical sources. Additionally, these models benefit from advanced optimization algorithms and larger parameter counts, enabling them to better interpret nuanced clinical questions and maintain logical coherence. In contrast, YouChat and Perplexity, while able to retrieve up-to-date information from the internet, may draw from sources of variable reliability, leading to inconsistencies or inaccuracies in the answers. Since the questions in our study were drawn from established pediatric guidelines and did not require the very latest research findings, the advantage of real-time search was minimized, and the strength of well-trained, static knowledge bases became more apparent. From another perspective, Retrieval Augmented Generation (RAG) seemed more capable of improving responses to professional questions. RAG allowed developers to control the quality of the data referenced by the models, overcoming the issue of outdated pre-trained model databases. Due to space and other constraints, further discussion on RAG was not included here.

This study utilized questions adapted from a professional pediatric publication to assess large language models’ responses related to pediatric fever. We acknowledge that such questions may not fully represent the natural language or phrasing typically used by caregivers in real-world settings. The professional phrasing of questions might have reduced the relatability and comprehension for non-medical participants, influencing their scoring behavior. This limitation could potentially affect the ecological validity of our findings. The large language models were developing rapidly, such as several GPT version updates by OpenAI during this study, making timeliness a limitation of our research. The increasing number of companies entering the large language model market and the variety of models made it impossible to cover all models, so this study could only include a part of these models, offering a reference for future research. Due to limitations such as funding and other objective conditions, our study was only able to use a small sample size (20 doctors and 120 relatives of pediatric patients) for the survey. To enhance the generalizability of our research findings, there was a need to expand the sample size in future studies. This study, limited to 30 questions on fever, required further exploration of large language models' capabilities in various medical issues.

The doctor-patient dual-layer study was the highlight of this study, introducing a novel approach to evaluate medical large language models, focusing on transitioning from a doctors' to a patients' perspective to provide understandable and central answers. We compared two internet-search-enabled models with two non-internet-search models, finding internet search reduced performance on the 30 subjective questions. Furthermore, we discussed potential methods to overcome outdated knowledge bases without reducing model performance.

## 5 Conclusions

Internet searches (YouChat and Perplexity) did not improve the ability of large language models to answer medical questions as expected. Patients lacked the ability to understand and analyze model responses due to a lack of professional knowledge and a lack of central points in model answers. When developing large language models for patient use, it's important to highlight the central points of the answers and ensure they are easily understandable.

## Data Availability

The original contributions presented in the study are included in the article/Supplementary Material, further inquiries can be directed to the corresponding author.
