# Supplementary figures and images for: Evaluating large language models in pediatric fever management: a two-layer study
Source: Front Digit Health. 2025 Sep 3;7:1610671. doi: 10.3389/fdgth.2025.1610671 (PMC12441047; doi:10.3389/fdgth.2025.1610671)

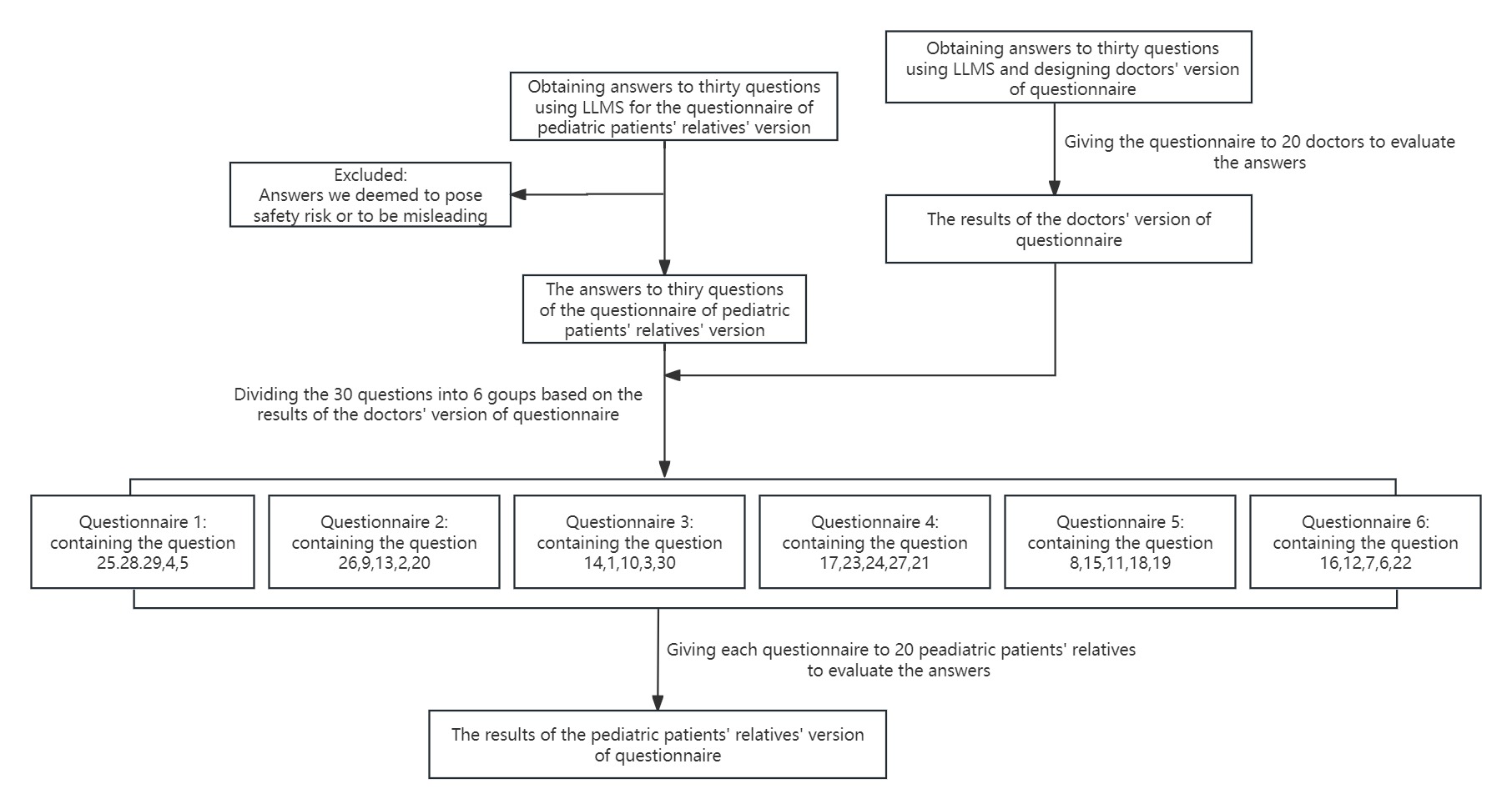

Supplement: Supplementary file 3 [file Image1.jpeg]
