# Supplementary material for: Evaluating large language models in pediatric fever management: a two-layer study
Source: Front Digit Health. 2025 Sep 3;7:1610671. doi: 10.3389/fdgth.2025.1610671 (PMC12441047; doi:10.3389/fdgth.2025.1610671)
Supplement: Supplementary file 4 [file Datasheet1.pdf]

Answers we deemed to pose safety risk or be misleading, questionnaires for pediatric patients' relatives and the flowchart.

ChatGPT3.5	Misleading answers	19
	Answers with safety risk	30
ChatGPT4.0	Misleading answers	None
	Answers with safety risk	28
Perplexity	Misleading answers	27
	Answers with safety risk	29
Youchat	Misleading answers	None
	Answers with safety risk	15
		28

Table S1. Answers we deemed to pose safety risk or be misleading.

Questionnarie	Questions in each Questionnaire				
1	25	29	28	4	5
2	26	9	13	2	20
3	14	1	10	3	30
4	17	23	24	27	21
5	8	15	11	18	19
6	16	12	7	6	22

Table S2. Questionnaires for pediatric patients' relatives.
